# Regulation of Photomorphogenic Development by Plant Phytochromes

**DOI:** 10.3390/ijms20246165

**Published:** 2019-12-06

**Authors:** Sharanya Tripathi, Quyen T. N. Hoang, Yun-Jeong Han, Jeong-Il Kim

**Affiliations:** Department of Integrative Food, Bioscience and Biotechnology, Chonnam National University, Gwangju 61186, Korea; tripathisharanya93@gmail.com (S.T.); ann.roll209@gmail.com (Q.T.N.H.); hanyj0716@gmail.com (Y.-J.H.)

**Keywords:** photomorphogenesis, phytochromes, light signaling, plant development, plant growth

## Abstract

Photomorphogenesis and skotomorphogenesis are two key events that control plant development, from seed germination to flowering and senescence. A group of wavelength-specific photoreceptors, E3 ubiquitin ligases, and various transcription factors work together to regulate these two critical processes. Phytochromes are the main photoreceptors in plants for perceiving red/far-red light and transducing the light signals to downstream factors that regulate the gene expression network for photomorphogenic development. In this review, we highlight key developmental stages in the life cycle of plants and how phytochromes and other components in the phytochrome signaling pathway play roles in plant growth and development.

## 1. Introduction

**Figure 1:**
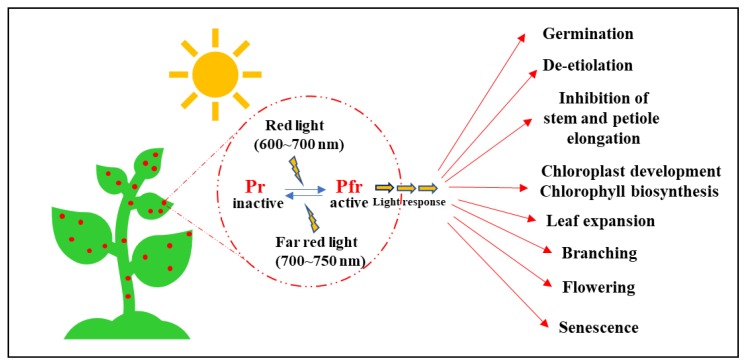
A schematic diagram depicting the involvement of phytochromes in different stages of photomorphogenesis. The red dots represent phytochromes that are present ubiquitously in plants. Inactive phytochrome (red light-absorbing Pr form) can be converted to active phytochrome (far-red light-absorbing Pfr form) by absorbing red light. The Pfr form can be converted back to the Pr form upon absorbing far-red light or in the dark (known as dark reversion, or more recently, thermal reversion). The active Pfr form regulates various photomorphogenic development through other downstream components of the phytochrome- mediated light signaling pathway.

Light is essential for plant growth and development, serving as an energy source for photosynthesis and as an environmental cue for photomorphogenesis (i.e., light-mediated development). Higher plants continuously adapt to their light environments to optimize their growth and development, which is monitored by various photoreceptors, including phytochromes [1]. As red (R) and far-red (FR) light-absorbing photoreceptors, phytochromes are dimeric chromoproteins with each monomer possessing a covalently linked open tetrapyrrole phytochromobilin as a chromophore. They are known to function as molecular switches with physiologically active FR light-absorbing (Pfr) and R light-absorbing (Pr) inactive forms. Upon absorbing R or FR light, the attached tetrapyrrole chromophore is photo-isomerized, inducing reversible conformational changes between the two forms of phytochromes [2]. Based on this system, phytochromes recognize different light information including light intensity and duration, transducing the signals to develop almost every step of the plant life cycle, from germination to flowering and senescence. In higher plants, phytochromes are encoded by small gene families; for example, dicotyledonous plants such as *Arabidopsis thaliana* have five members, phytochrome A (phyA) to phytochrome E (phyE), and monocotyledonous plants such as *Oryza sativa* have three members (phyA to phyC) [3]. Furthermore, these phytochromes are classified into light-stable type I (phyA) and light-labile type II (phyB to phyE) species [4]. It is well known that phyA regulates FR light signaling, while phyB to phyE regulate R light signaling [5]. These members have partially redundant yet distinctive functions throughout the lifespan of a plant, starting from seed dormancy and germination to seedling de-etiolation [6,7], photomorphogenesis [8], reproductive transition [9], and senescence [10] (Figure 1). In 1952, one classical experiment demonstrated that R light exposure increases the seed germination of lettuce (*Lactuca sativa* L.) from 8.5% to 98% [11]. Recently, similar results have been demonstrated in the model plant, *A. thaliana* [6,7]. When seeds are buried under soil, seedlings show an etiolated growth pattern (i.e., skotomorphogenesis), in which hypocotyls elongate and cotyledons fold to form hooks until they reach up to the surface of the soil for sunlight. Upon exposure to sunlight, hypocotyl elongation stops, cotyledons open, and functional chloroplasts develop, leading to photomorphogenic development mediated by different phytochromes [9]. Roles of phytochromes do not stop here. During plant growth and development, phytochromes keep working in elongating branches towards light when shaded by neighboring foliage, transiting from the vegetative phase to the reproductive phase at the appropriate time [12], and senescence [10]. Notably, the photo-reversibility of phytochromes by R and FR light is only observed in low fluence responses (LFR), where phyB plays the dominant role [13], with redundant functions of phyD and phyE [14]. While phyB to phyE act in LFR, phyA has been found to work under very low fluence responses (VLFR) and FR-high irradiance responses (FR-HIR) [5].

**Figure 2:**
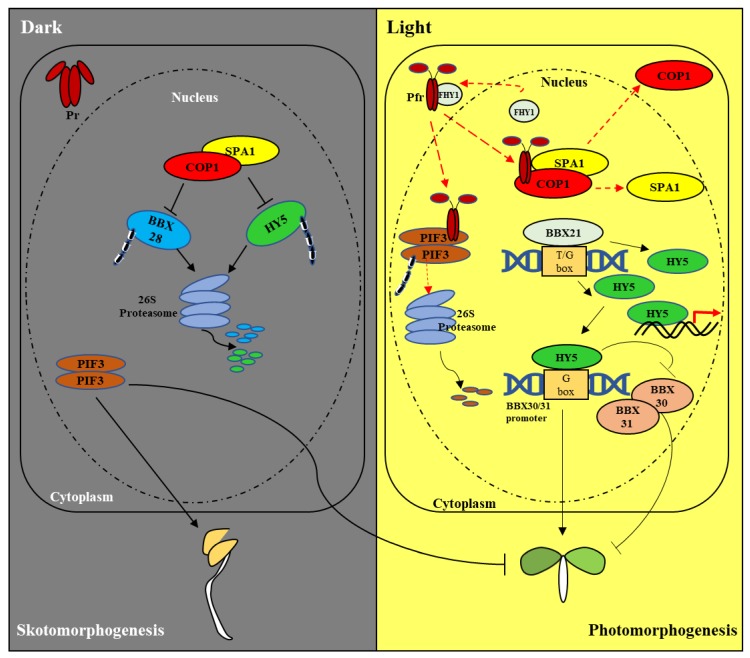
A simplified view of the phytochrome-mediated light signaling pathway in *A. thaliana*. For simplicity, PIF3 (phytochrome interacting factor 3) was used as a representative of PIFs and SPA1 (suppressor of *phyA-105* 1) as a representative of the SPA proteins (SPA1 to SPA4). In the dark (left panel), phytochromes are synthesized as the inactive Pr form, remaining in the cytoplasm. Meanwhile, PIF proteins accumulate in the nucleus and negatively regulate the expression of genes involved in photomorphogenesis (shown as a T-headed line), allowing skotomorphogenesis (shown as an arrow-headed line). In addition, the COP1(constitutive photomorphogenesis protein 1)/SPA1 complex degrades HY5 (elongated hypocotyl 5) and BBX28 (B-box domain protein 28) via the ubiquitin/26S proteasome-mediated pathway to inhibit photomorphogenesis. Under light condition (right panel), photoactivated phytochromes (Pfr) accumulate in the nucleus (For phyA, FHY1 (far-red elongated hypocotyl 1) and FHL (FHY1-like) are the facilitators for the nuclear localization. Here, only FHY1 is shown for simplicity). In the nucleus, the Pfr form interacts with downstream signaling components such as PIF3 and the COP1/SPA1 complex. Phytochromes inactivate PIF3 via the 26S proteasome-mediated degradation pathway, and also inactivate the COP1/SPA1 complex by inducing dissociation, in which COP1 is subsequently exported to the cytoplasm. Among B-box proteins, BBX21 binds to the T/G box region of *HY5* promoter and upregulates its expression. Thus, the inactivation of the COP1/SPA1 complex and BBX21 function contribute to the accumulation of HY5. In turn, HY5 induces the expression of light-responsive genes for photomorphogenesis (red arrow), and also suppresses BBX30 and BBX31, the negative regulators of photomorphogenesis, all of which promote photomorphogenic development.

Phytochromes are synthesized in the cytosol as the Pr form and converted to the Pfr form upon absorbing R light. This photoactivated Pfr form translocates from the cytosol to the nucleus, where they regulate the transcription of light-responsive genes through several transcription factors such as PIFs (phytochrome-interacting factors) [8,15]. The active, thermally unstable Pfr can be converted back to the inactive Pr form by absorbing FR light or in a light-independent process called dark reversion or thermal reversion [16,17]. PIFs are a family of basic helix-loop-helix (bHLH) transcription factors that have many roles including seedling etiolation. For example, the dark-grown Arabidopsis quadruple *pifq* (*pif1pif3pif4pif5*) mutant showed shortened hypocotyls and opened cotyledons comparable to light-grown wild type seedlings [18]. Phytochromes are known to phosphorylate PIFs, which induces their degradation via the 26S proteasome-mediated pathway [19]. On the other hand, PIFs also control phyB abundance in the nucleus and mediate its degradation [20]. COP1 (Constitutive photomorphogenesis protein 1), an E3 ubiquitin ligase, targets several positive regulators of photomorphogenesis for their degradation in the dark, which include HY5 (elongated hypocotyl 5), HYH (HY5-homolog), HFR1 (long hypocotyl in far-red), and LAF1 (long after far-red light 1) [21]. Upon light illumination, COP1 is inactivated via multiple regulatory mechanisms, releasing the positive photomorphogenesis regulators to function [22,23]. Recent findings have reported that B-box domain proteins (BBXs) function as positive and negative regulators of photomorphogenesis in the HY5-mediated pathway [24,25]. BBX28, BBX30, and BBX31 are negative regulators of photomorphogenesis, in which BBX28 works upstream of HY5, and BBX30 and BBX31 work downstream of HY5. In the dark, COP1 degrades both BBX28 and HY5 by the 26S proteasome-mediated pathway. Upon light illumination, with inactivation of COP1, BBX28 is accumulated and interacts with HY5, which inhibits HY5 binding to its target sites and thus represses HY5 activity. On the other hand, BBX20, BBX21, BBX22, and BBX23 upregulate *HY5* gene expression and lead to an increase in the HY5 level, which can bypass the suppression of BBX28 on transcriptional activity, promoting photomorphogenesis [26,27]. In the nucleus, activated phytochromes directly interact with SPA (suppressor of *phyA-105*), and induce the dissociation of COP/SPA complexes to promote photomorphogenesis [28]. Therefore, phytochromes mediate photomorphogenic development by inhibiting the function of negative regulators of photomorphogenesis, i.e., PIFs and COP1/SPA complexes. The former is degraded in the presence of phytochromes, and the latter is dissociated by binding with phytochromes (Figure 2).

## 2. Roles of Phytochromes in the Regulation of Seedling Establishment

### 2.1. Seed Germination

The photomorphogenic development of plants commences with seed germination. The promotion of germination is mediated by phytochromes [29] and levels of two hormones, abscisic acid (ABA), and gibberellic acid (GA) that function antagonistically [30]. ABA plays important roles in seed dormancy under unfavorable conditions, whereas GA promotes seed germination when environmental conditions are favorable. In dicots, each phytochrome member (phyA to phyE) provides seeds the ability to respond and adjust the timing and place of germination to different environmental cues [29]. Arabidopsis seeds, when properly sensitized to light, germinate after irradiation with VLFR through phyA signaling, and secondarily through phyD and phyE [29], whereas seeds less sensitive to light require a higher photon fluence (LFR) to germinate through phyB [31]. So far, information concerning the molecular basis of phyB-mediated germination is better understood than that on phyA-mediated germination [32,33]. Light-dependent activation of phyB modulates ABA and GA signaling and metabolism. PIF1 (also known as PIL5 or PIF3-like 5), RVE1 (reveille 1), and RVE2 are the repressors of germination [33,34]. PIF1 is known to repress seed germination either directly or indirectly through DELLA proteins, such as GAI (GA-insensitive) and RGA (repressor of GA), when phyB is inactive [33,35]. PIF1 was initially known to repress seed germination in the dark [36]. Under the light condition, photoactivated phytochromes translocate to the nucleus and degrade PIF1 protein via the ubiquitin/proteasome system, which has been suggested to act as the regulatory mechanism of phytochromes in the promotion of seed germination [37].

To better understand the regulation of seed germination, we need to ascertain the function of PIF1 and its regulation. PIF1 directly upregulates the expression of *SOM* (SOMNUS), *ABI3* (abscisic acid insensitive 3), and other repressors of seed germination [33,38]. In addition, DAG1 (DOF affecting germination 1) was shown to repress germination downstream to PIF1 by directly repressing *GA3ox1* [32]. PIF1 is also known to be regulated by de-etiolated 1 (DET1) that functions upstream of HFR1, a key positive transcriptional regulator of seed germination [39]. A KELCH F-box protein, CTG10 (cold temperature germinating 10) can bind to PIF1, negatively influencing PIF1 stability, stimulating the completion of germination [40]. In addition to HFR1 and CTG10, two jumonji C-domain containing histone demethylases, JMJ20 and JMJ22, acting redundantly as positive regulators of germination through the interaction with SOM, have been discovered. When phyB is inactive, *JMJ20*/*JMJ22* are directly repressed by SOM. Following phyB activation, *JMJ20*/*JMJ22* are de-repressed, resulting in increased GA levels through the removal of repressive histone methylation at *GA3ox1*/*GA3ox2*, which in turn promotes seed germination [6]. Moreover, several conspicuous DELLAs (*GAI*, *RGA*, and *RGL2*) have been found to be downregulated by phyA [31]. In addition, the expression of auxin transport (*PIN1*, *PIN2*, *PIN7*), signaling (*RED1*, *AXR4*, *AXR1*, *Saur-LIKE*, *AFR18*, *GH3.6*), and metabolic (*NIT3*, *SUR1*, *CYP79B2*) genes is positively regulated by the function of phyA. Moreover, ABA metabolic genes have been found to be downregulated by phyA function, whereas the expression of key regulators of cell wall expansion, *EXPANSINs* (*EXP1*, *EXP2*, and *EXP10*), has been shown to be induced [31]. A recent study further demonstrated that upon seed imbibition, PIF1 was re-accumulated quickly and stabilized. However, the phyA response under a canopy can trigger polyamine levels in seeds until it bypasses PIF1 to allow seeds to germinate [41]. Therefore, recent studies suggest that phytochromes play important roles in seed germination via PIF1 regulation and also via PIF1-independent pathways that are involved in hormone signaling.

### 2.2. De-Etiolation

Under the soil, germinating seedlings undergo etiolation with long hypocotyls and closed cotyledons, lacking chlorophylls and functional chloroplasts (i.e., skotomorphogenesis). Upon emerging from the soil and reaching light, the etiolated seedlings undergo de-etiolation, which includes cotyledon opening, chlorophyll biosynthesis, chloroplast development, and subsequently autotrophic growth (i.e., photomorphogenesis) [42]. Phytochromes and four PIF members (PIF1, PIF3, PIF4, PIF5) play a central role in these etiolation and de-etiolation events, along with other regulators. Upon FR and R light exposure, phyA and phyB undergo a nuclear translocation, which leads to phosphorylation and rapid degradation of PIFs, the negative transcription regulators in photomorphogenic development [43]. Thus, the removal of functional PIFs releases the genome-wide suppression of transcription, promoting photomorphogenesis [44]. It is also notable that a VQ29 (VQ motif-containing protein 29) has been shown to physically interact with PIF1, and VQ29-PIF1 directly binds to the promoter of a cell elongation-related gene, *XTR7* (xyloglucan endotransglycosylase 7), for its expression [45]. In addition to PIFs, COP/DET/FUS complexes are important for the seedling de-etiolation process [46]. COP1 and its complex with SPAs (SPA1 to SPA4) negatively regulate the levels of several photomorphogenesis-promoting proteins, including phyA, CRY2 (cryptochrome 2), HY5, HYH, HFR1, and LAF1 [43,46]. Particularly, HY5 directly binds to both C/G box and G box in the promoter of a positive regulator of de-etiolation, HTL (hypersensitive to light), which regulates phytochrome- and cryptochrome-mediated de-etiolation responses [43]. In addition, PAR1 (phytochrome rapidly regulated 1) and PAR2 play a role in seedling de-etiolation, in a separate pathway from HY5 and HFR1 under different light conditions [47]. However, de-etiolation in monocots has not been well studied. Notably, OsPIL15, a member of the rice PIF family, has been reported to repress etiolated seedling growth in rice; however, it acts antagonistically to Arabidopsis PIFs [48].

## 3. Plant Architecture Regulated by Phytochromes

### 3.1. Shoot-Root Development and Branching

**Figure 3:**
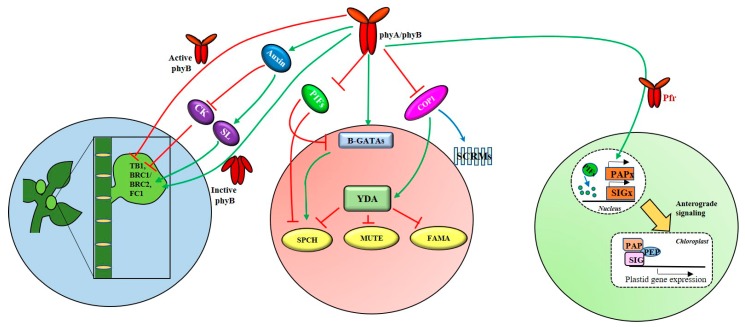
A simplified view of signaling pathways involved in phytochrome-mediated shoot branching, stomatal development, and chloroplast development. The green arrows represent positive regulation, red T-headed lines represent the inhibition of gene function and blue arrows represent degradation.

Plants dynamically adjust their architecture to optimize growth and development under fluctuating light conditions, which include the deceleration of hypocotyl elongation, cotyledon opening, leaf greening, root elongation, lateral root proliferation, and root tropisms [49,50]. Root elongation and root hair formation are mediated by phyA and phyB under FR/blue and R light conditions, respectively. Furthermore, lateral root production is regulated by phytochromes, in which phyA, phyB, and phyE stimulate lateral root production, while phyD inhibits the lateral root development in Arabidopsis [49]. In monocots, phyA and phyB are also known to be responsible for light-regulated inhibition of the seminal root elongation [49,51]. In fact, photoreceptors occur ubiquitously over the plant body including roots, although they are most abundant in shoots [52]. A recent study has demonstrated that light is transmitted through the stem to the root, and that the stem-piped light activates root phyB [50]. Reportedly, the photoactivated phyB localized to the nucleus of root cells induced transcription of *HY5* and stabilization of its protein product. In shoots, COP1 regulates shoot to root auxin transport by controlling the transcription of the auxin efflux carrier genes, *PIN1* and *PIN2* [53]. Shoot branching is another important aspect that contributes to plant architecture. Branching is the result of several inter-related developmental programs beginning with axillary meristem initiation, formation of the axillary bud, initiation of bud outgrowth, and then branch elongation [54]. Regulation of BRC1 (Branched 1) is the central hub for many shoot branching-related mechanisms and the expression of *BRC1* is directly or indirectly regulated by phyB (Figure 3) [55,56]. *A. thaliana* comprises two *BRANCHED* genes, *BRC1* and *BRC2*, which encode TCP transcription factors and are closely related to *TB1* (teosinte branched 1) in maize and *FC1* (fine culm 1) in rice [57]. *TB1* in maize, *OsTB1/FC1* in rice, and *SbTB1* in sorghum are known to promote bud arrest locally [57,58]. It has been reported that active phyB suppressed *SbTB1* and *AtBRC1* in sorghum and Arabidopsis, respectively, leading to high branching, whereas inactive phyB (under low R:FR) increased these gene expressions and repressed branching [55,56]. In addition, phyB regulates shoot branching through the components involved in the auxin signaling pathway [54]. Furthermore, it has been reported that *phyB* mutants had reduced auxin signaling resulting from AXR1 (auxin-resistant 1) deficiency and showed high branching, which indicates auxin signaling downstream to phyB [59]. Auxin inhibits bud outgrowth through the promotion of systemic and local strigolactone (SL) synthesis by upregulating SL biosynthesis genes, *MAXs* (more axillary growth) in Arabidopsis. Furthermore, SL upregulates *BRC1* expression and inhibits branching [57]. In parallel, auxin negatively regulates systemic and local cytokinin (CK) levels in the stem and buds. CK is known to be a powerful repressor of expression, where a decrease in the CK level elevates *BRC1/TB1/FC1* expression and inhibits bud outgrowth [57].

### 3.2. Stomata Development

The production of stomata, which mediates gas and water vapor exchange between plant and the environment, is regulated by plant hormones and signal peptides such as EPFs (epidermal patterning factors), along with environmental stimuli. The stomatal development process starts with cell division and differentiation of MMCs (meristemoid mother cells), which further generate small meristemoid, a large SLGC (stomatal lineage ground cell), and GMC (guard mother cell). This cell fate transition process involves bHLH transcription factors such as SPCH (SPEECHLESS), MUTE, FAMA in partnership with their heterodimerization partners, SCREAM 1 (SCRM1) and SCRM2, which act together as positive regulators for stomatal development to consecutively promote initiation of asymmetric division, proliferation of precursor cells, and the differentiation of guard cells [60,61]. In addition, several negative regulators play critical roles in the regulation of development and patterning of stomata in Arabidopsis, including ER (erecta) family of LRR (leucine-rich repeat) receptor-like kinases (ER, ERL1, ERL2), the LRR receptor-like protein TMM (too many mouths), their putative ligand EPF1 (epidermal patterning factor 1), a subtilisin protease SDD1 (stomatal density and distribution 1), and MAPK (mitogen-activated protein kinase) signaling components such as YDA (yoda), MKK4/5, and MPK3/6 [61]. Light is one of the stimuli, perceived by phytochromes and other photoreceptors, which then regulates stomatal patterning and development through its downstream signaling components (Figure 3). The components of light signaling (such as PIFs and COP1) and those of the stomata developmental pathway work together to regulate the whole process for stomatal development. Under FR and R/white light conditions, phyA and phyB regulate stomatal development, mainly through negatively regulating COP1 and PIF4 [61,62,63]. COP1 regulated stomatal development upstream of YDA, and a loss-of-function mutation of either COP1 or YDA developed the stomata constitutively and produced in a cluster, both in light and dark conditions [61,64]. In the dark, when COP1 is stable, it degrades SCRM1 and SCRM2, independently of the YDA pathway [65]. Another report indicated that a light-induced GATA transcription factor of subfamily-B (B-GATA) promotes stomatal development in hypocotyls of *A. thaliana* [66,67]. B-GATA works upstream of SPCH/MUTE/FAMA, but downstream of and suppressed by PIFs [68]. B-GATAs promote SPCH expression by directly binding to its promoter region [66]. Arabidopsis PIF4 binds and suppresses SPCH, a master regulator of stomatal lineage initiation under high temperature [69]. Since phyB negatively regulates *EXPANSIN* and *ERECTA* gene expression in leaves, the loss of phyB function results in greater epidermal cell expansion, accompanied by a reduced stomatal density and transpiration rate. This explains the reduced water loss and improved drought tolerance in rice and tomato [70,71]. It is also notable that *Zea mays* PIF3 (ZmPIF3) and ZmPIF1 in rice and Arabidopsis have been reported to achieve increased sensitivity to ABA, resulting in more water-saving and drought resistance [72,73].

### 3.3. Chloroplast Development

In plants, the chloroplasts are crucial for their growth. The plastids not only function in the performance of photosynthesis, but also sense environmental stimuli [74]. In addition, many biochemical processes take place in chloroplasts, such as biosynthesis of pigments, lipids, and hormones. Chloroplasts have their own genome and RNA polymerase. Plastids of vascular plants contain two types of RNA polymerases, a plastid-encoded, bacterial-type multi-subunit RNA polymerase called PEP (plastid-encoded polymerase) and a nuclear-encoded, phage-type single-subunit RNA polymerase called NEP (nuclear-encoded polymerase) [75]. NEP functions in transcribing mainly plastid-encoded housekeeping genes, plastid ribosomal RNAs, and subunits of PEP. In contrast, PEP transcribes mostly photosynthesis-related and tRNA genes. The PEP enzyme needs to interact with nuclear-encoded SIGs (sigma factors) for proper promoter recognition and also with PAPs (PEP-associated proteins) for chloroplast biogenesis [75].

The light signal is important for the biogenesis and development of chloroplasts (Figure 3). To explain how the light signal is transferred from the nucleus to chloroplasts, an anterograde pathway has been proposed. This pathway shows how downstream signals of phyB can regulate the chloroplast gene expression. Upon light activation, the active phytochromes trigger light-dependent PEP assembly by forming photobodies, and destabilizing PIFs [76]. Following the degradation of PIFs, repression in the promoters of *PhANGs* (photosynthesis-associated nuclear-encoded genes) is released, allowing SIGs and PAPs to be expressed. Next, SIGs and PAPs form a complex with PEP for the transcription of *PhANGs*, which allows anterograde signaling in plastid gene expression [76,77].

## 4. Effect of Light Quality and Quantity on Plant Vegetative Growth

Plants adopt two strategies to deal with shade conditions, shade avoidance, and shade tolerance. Under shaded conditions, most species exhibit shade avoidance syndrome (SAS) to escape shade. However, small plants from forest understories cannot outgrow surrounding trees and hence adopt a tolerance response [78]. Reportedly, phyB is the most important photoreceptor in the shade avoidance responses, along with the redundant roles of phyD and phyE [79,80]. In contrast, phyA plays an important role in shade tolerance [78,81]. Under low R:FR ratio condition, the Pfr form converts to the Pr form, leading to re-accumulation and stabilization of PIFs. Next, the accumulated PIFs promote stem elongation by binding to G-box motifs in a broad range of target genes. In addition, hypocotyl elongation in low R:FR involves rapid auxin biosynthesis through TAA1 (tryptophan aminotransferase of Arabidopsis 1) [79]. PIF1, PIF5, and PIF7 play major roles in this process, which regulate cell elongation, in part, by upregulating the transcription of YUCCA enzymes that control the rate-limiting step of a major auxin biosynthesis pathway [78,79]. This has increased the expression and re-localization of the auxin efflux regulator PIN3 to supply auxin to the hypocotyl epidermis. Moreover, COP1/SPA complexes degrade the negative regulators of PIFs, such as HY5 and HFR1, further stabilizing PIFs [79,82]. Under deep shade (i.e., very low R:FR ration condition), phyA accumulates in the nucleus, releasing AUX/IAA (auxin/indole-3-acetic acid) from SCF^TIR1^ auxin receptor complex, resulting in the negative regulation of auxin signaling to afford plants tolerance to deep shade [81].

## 5. Flowering and Senescence

### 5.1. Flowering

Flowering during a season is the most critical event for reproductive success. Hence, when to initiate flowering is an important decision in the life cycle of a plant [83]. During flowering, light is a major stimulus, which determines the timing for the transition from the vegetative to the reproductive phase. In addition, it regulates the period of flowering, called photoperiod. Based on the flowering pattern of plants in response to the photoperiod, they are categorized into long day (LD), short day (SD), and day-neutral plants. CONSTANS (CO), a zinc finger protein is a central stabilizer in the photoperiod, although the *CO* transcription pattern remains unaltered with changes in the photoperiod. Rhythmically, CO activates FT (flowering locus T) in the leaf, and then, FT travels a long distance through the phloem to the shoot apex where it initiates flowering [84]. In the dark, COP1-SPA complexes suppress *CO* expression followed by the suppression of *FT* expression. Photoreceptors play a pivotal role in this flowering event. Notably, phyA is the master regulator for morning activated genes, especially under the SD condition, whose expression is directly controlled through interaction with PIF4 and PIF5 [85]. Photoactivated phyA and phyB compete with COP1 for binding to SPAs, which leads to inactivation of COP1-SPA complexes [86]. On the other hand, phyB destabilizes CO in the morning through another E3 ubiquitin ligase, HOS1 (high expression of osmotically responsive gene 1). Here, PHL (phytochrome-dependent late-flowering) physically interacts with phyB, which interferes with phyB-mediated destabilization of CO in the afternoon [84,87]. In fact, phyA and phyB antagonistically regulate the transcription of *CO*, the key regulator of flowering. In response to R light, phyB downregulates *CO* transcription with the help of other factors such as PET1 (phytochrome and flowering time 1), whereas phyA upregulates *CO* transcription [87]. It should be noted that the role of phytochromes in flowering varies with plant species. Loss-of-function phyA mutations in Arabidopsis and rice do not influence flowering during the inductive photoperiod. However, garden pea showed a 20-day delay in flowering under both SD and LD conditions [88]. On the other hand, phyB mutants showed early flowering in a range of species including Arabidopsis, rice, pea, and sorghum (see references in [88]). In the case of phyC, loss-of-function mutations in both Arabidopsis (a LD plant) and rice (a SD plant) showed slightly accelerated flowering under the SD condition, suggesting phyC as a weak floral repressor [88]. However, Arabidopsis phyC is non-functional in the absence of other phytochromes [89]. In other plants such as Brachypodium and wheat, phyC plays a major role in floral initiation, with mutations demonstrating extremely delayed flowering [88,90]. In barley, phyC is also reported to control photoperiod sensitivity under a long photoperiod [91]. Moreover, not only flowering, phyA and phyB synergistically regulate anther and pollen development in rice, as suggested by a recent study [3].

### 5.2. Senescence

Lastly, plants show senescence to shed photosynthetically inefficient leaves by mainly two ways. In one, mainly observed in deciduous forests, trees shed their leaves in a relatively narrow time frame, leaving the canopy leafless. In the second pathway, plants shed leaves regularly, with the oldest ones being shed first, continuing with the growth and development of newer leaves [92]. Senescence starts with light deprivation or in darkness, which involves transcriptional reprogramming to disassemble cellular components and remobilize nutrients [9]. For decades, phytochromes have been known to play a role in dark-induced senescence. For example, back in 1971, a study on *Marchantia* (a liverwort) showed that 5 min R light pulse per day inhibited dark-induced senescence and could be reversed by 10 min irradiation of FR light [93]. Several experiments in other species such as barley, cucumber, tomato, and mustard also showed comparable results [9]. These experiments supported the role of phytochromes in dark-induced senescence, especially in terms of chlorophyll degradation. Phytochromes are the negative regulators of dark-induced senescence, modulating a molecular feed-forward loop with PIFs [9]. PIF3, PIF4, and PIF5 are essential for dark-induced senescence [9,94,95]. ELF3 (early flowering 3) and phyB inhibit senescence by suppressing the function of PIFs at the transcriptional or post-transcriptional level, respectively, in a light-dependent manner [9]. In addition, two major senescence-promoting hormones, ethylene and ABA, activate the expression of *EIN3* (an ethylene-related transcription factor), *ABI5* (an ABA-related transcription factor), and *EEL* (Enhanced EM Level, a transcription factor homologous to ABI5) with the help of PIF4/PIF5. Furthermore, these transcription factors activate the major senescence pathway inducing the NAC transcription factor, ORE1 (oresara 1) [92]. Additionally, a couple of insights have demonstrated that PIF4 and PIF5 regulate ethylene signaling and age-induced leaf senescence by direct activation of *NYE1* (a chlorophyll degeneration regulatory gene) and suppression of *GLK2* (golden2-like 2), a gene that maintains chloroplast activity [94,95]. PIF5 physically interacts with the G-box motifs in the promoters of several chlorophyll breakdown genes such as *SGR*, *NYC1*, and *ORE1*, stimulating their expression [95]. Forest floors or densely populated plantations are generally enriched with FR light, and phyA plays main roles under these conditions. Thus, phyA functions to maintain leaf chlorophyll content in response to a partial shade condition, allowing leaves to adjust the photosynthetic machinery under very low irradiance conditions. This helps maintain a positive carbon balance and represses leaf senescence under prolonged shade conditions [96]. Furthermore, a recent study reported that phyA and phyB work antagonistically to regulate FR enhanced senescence by WRKY6 [97,98]. Under FR light, phyA inhibited leaf senescence by repressing *SAG* gene expression, whereas phyB promoted leaf senescence. When phytochromes sense FR light, the signal is transferred to the leaf senescence pathway via WRKY6 that binds directly to the promoter of *SIRK* (senescence-induced receptor-like protein kinase) and induces the gene expression. Thus, WRKY6 acts as a positive regulator for leaf senescence, and regulates dark-induced senescence by upregulating *SAG* expression [98]. Another report indicates a possible role of HY5 in the leaf senescence pathway by demonstrating that *WRKY6* and *SAG29* are the putative targets of HY5 [99]. Based on the mentioned studies above, it appears that phytochromes and their downstream components (such as PIFs and HY5) might be working with senescence-related and hormone-related genes in a complex pathway. Therefore, light-dependent leaf senescence needs further investigation to understand the roles of each involved component.

## 6. Conclusions and Perspectives

Recently, we reviewed the regulatory mechanisms for phytochrome-mediated light signaling pathway [100], based on our results that phytochromes might function as a protein kinase in plants [19]. Here, we tried to illustrate the roles of phytochromes and their downstream signaling components during plant growth and development. The signaling network for phytochrome-mediated photomorphogenic development is divided into four parts. The first part is the light absorption and conformational changes, the second part is the interaction of phytochromes with various downstream components and signaling initiation, the third part is the regulation of signaling via ubiquitin/26S proteasome-mediated proteolysis and signal integration, and the final part is the regulatory gene expression of light-responsive genes. In genome-wide expression data, approximately 2,500 genes which is ~ 10% of Arabidopsis genome were regulated by phytochrome under prolonged light exposure, where ~ 80% of the total light-responsive genes were induced, with ~ 20% being repressed [98]. Most of these genes are involved in the plant transition from heterotrophic to autotrophic life, which includes photosynthesis, hormone pathways, and metabolic pathways. Thus, the function of phytochromes could be the transcriptional regulation of genes involved in photomorphogenesis via negative (PIFs, COP1/SPA complexes, etc.) or positive (HY5, HFR1, etc.) transcription factors [15]. Moreover, phytochromes are also involved in other physiological processes including stress responses, defense, stomatal opening, and the relative oxygen species (ROS) pathway [101,102,103,104]. In addition, phytochromes interact with other photoreceptors to regulate these processes, including cryptochromes and phototropins. Here, we have summarized the signaling components involved in phytochrome-mediated photomorphogenic development (Table 1).

Although our understanding of phytochrome-mediated photomorphogenic development has significantly improved in recent years, the available knowledge is limited and fragmented. For example, most studies were conducted in Arabidopsis, so phytochrome-mediated signaling in monocots is yet to be elucidated. As an example, while phyB is known to repress flowering in *A. thaliana*, phyB promotes flowering in rice and wheat, via combined function with phyC [105,106]. In addition to the phytochrome functional diversity between dicots and monocots, phytochromes are also functionally diverse among monocots. For example, phyC promotes flowering in Brachypodium, whereas phyC delays heading dates in rice under LD condition [88,107]. Therefore, thorough and detailed research is imperative to understand phytochrome-mediated light signaling and photomorphogenic development in monocots. More recently, phyB has been reported as a thermosensor in plants [16,17]. Considering global warming due to climate changes, plant thermomorphogenesis will be an important research topic in the near future to improve crops that are growing under high-temperature challenges. Regarding this issue, a possible research direction would be exploring the involvement of other phytochromes in temperature sensing. Notably, some phytochromes can only form homodimer only, whereas others can heterodimerize with other phytochromes. Considering that different phytochromes seem to play different roles or cooperate in plant development, it is necessary to investigate the possibility that the functional differences of phytochromes or their co-function could be linked to their dimerization state.

## Figures and Tables

**Table 1:** Signaling components involved in phytochrome-mediated photomorphogenesis.

Component	Characteristics	Function	Signaling	Reference
PIF1/PIL5	bHLH TF ^1^	Negative regulator of seed germination	phyA and phyB signaling	[36,39]
RVE1 and RVE2	Myb-like TF	Activator of seed dormancy	phyB signaling	[34]
HFR1	bHLH TF, but no direct binding to DNA	Positive regulator of seed germination and seedling de-etiolation	phyA and phyB signaling	[39]
SOM	CCCH-type zinc finger protein	Negative regulator of seed germination	phyA and phyB signaling	[6,31]
JMJ20 and JMJ22	Histone demethylase	Positive regulator of seed germination	phyB signaling	[6]
ABI4	Ethylene-responsive TF	Regulates ABA signaling during seed germination, shoot branching	phyA signaling and PIL5 signaling	[31,54]
AXR1	NEDD8-activating E1 regulatory subunit	Regulates auxin signaling during seed germination	phyA signaling	[31]
AXR4	Auxin-responsive protein	Regulates auxin signaling during seed germination	phyA signaling	[31]
PIN7	Auxin efflux carrier component 7	Mediates auxin gradient during seed germination	phyA signaling	[31]
PIN1 and PIN2	Auxin efflux carrier component 1 and 2	Mediates auxin gradient during seed germination, root elongation	phyA signaling and PIL5 signaling	[31,53]
PIF3, PIF4, and PIF5	bHLH TF	Negative regulators of seedling de-etiolation	phyA and phyB signaling	[43]
COP/DET /FUS	E3 ubiquitin ligase	negative regulator of seedling de-etiolation	phyA and phyB signaling	[46]
HY5 and HYH	bZIP TF ^2^	Positive regulator of seedling de-etiolation	phyA and phyB signaling	[43,46]
LAF1	R2R3 MYB-like TF	Positive regulator of seedling de-etiolation	phyA signaling	[43,46]
HTL	α/β fold protein	Works downstream to HY5	phyA and phyB signaling	[43]
PAR1 and PAR2	bHLH TF	Works downstream to COP1	phyA and phyB signaling	[47]
Rice PIL15	bZIP TF	Positive regulator of seedling de-etiolation in rice	phyB signaling	[48]
BRC1 and BRC2	TCP domain group proteins	Inhibit shoot branching under low R:FR	phyB signaling	[54]
MAX2	F-box leucine-rich protein	Inhibit shoot branching	phyB signaling	[54]
MAX4	Carotenoid cleavage dioxygenase	Inhibit shoot branching	phyB signaling	[54]
YDA	Mitogen-activated protein kinase kinase kinase (MAPKKK)	Regulates stomatal development	COP1 signaling	[64]
SPCH	bHLH TF	Regulates stomatal development	PIF4 signaling	[69]
B-GATA	GATA TF	Regulates stomatal development	PIF signaling	[66]
EXPANSIN	α/β fold protein	Negative regulator of stomatal development	phyB signaling	[70]
ERECTA	LRR receptor-like kinase	Negative regulator of stomatal development	phyB signaling	[70]
SIG2 and SIG6	Sigma factors	Regulation expression of photosynthetic genes, and chloroplast development	phyA and phyB signaling	[76,77]
TAA1	Aminotransferase	Regulation of auxin biosynthesis during hypocotyl elongation	PIF signaling	[79]
YUCCA	Flavin monooxygenase	Regulation of auxin biosynthesis during hypocotyl elongation	PIF signaling	[78,79]
AUX/IAA	Repression complex in auxin signaling	Auxin signaling during seedling etiolation	phyA signaling	[81]
CONSTANS	Zinc finger TF	Regulation of photoperiodic flowering	phyA and phyB signaling	[87]
PFT1	Mediator complex subunit 25 (MED25)	Promotion of flowering	phyB signaling	[87]
PHL	Glutamine-rich nuclear protein	Promotion of flowering	phyB signaling	[87]
ELF3	TF in circadian clock input pathway	Delays senescence	phyB and PIF signaling	[10]
EIN3	TF in ethylene signaling pathway	Regulates ethylene signaling during senescence	PIF signaling	[10]
ABI5 and EEL	bZIP TF	Regulates ABA signaling during senescence	PIF signaling	[10]
NYE1	Chloroplast protein	Regulation of chlorophyll degradation	PIF signaling	[95]
SGR	Chloroplast protein	Regulation of chlorophyll degradation	PIF signaling	[96]
ORE1	NAC TF	Positive control of senescence	PIF signaling	[96]
WRKY6	Zinc finger TF	Regulator of senescence	phyA and phyB signaling	[98]

^1^ bHLH TF: Basic helix-loop-helix transcription factor; ^2^ bZIP TF: Basic leucine zipper TF.
